# Ethiodized oil as an imaging biomarker after conventional transarterial chemoembolization

**DOI:** 10.1007/s00330-023-10326-7

**Published:** 2023-11-06

**Authors:** Mishal Mendiratta-Lala, Anum Aslam, Harrison X. Bai, Julius Chapiro, Thiery De Baere, Shiro Miyayama, Victoria Chernyak, Osamu Matsui, Valerie Vilgrain, Nicholas Fidelman

**Affiliations:** 1 Department of Radiology, University of Michigan Medicine, 1500 E Medical Center Dr., UH B2 A209R, Ann Arbor, MI 48109 USA; 2 Department of Radiology and Radiological Sciences, John Hopkins University, 601 N Caroline St, Baltimore, MD 21287 USA; 3 Department of Radiology & Biomedical Imaging Yale University School of Medicine, 300 Cedar Street - TAC N312A, New Haven, CT 06520 USA; 4 Gustave Roussy University of Paris Saclay, Villejuif, France; 5 Interventional Radiology, Gustave Roussy Cancer Center, Villejuif, France; 6 Département d’Anesthésie, Chirurgie et Imagerie Interventionnelle, Gustave Roussy Cancer Center, Villejuif, France; 7 Department of Diagnostic Radiology, Fukui-ken Saiseikai Hospital 7-1, Funabashi, Wadanaka-cho, Fukui, 918-8503 Japan; 8 Department of Radiology, Memorial Sloan Kettering Cancer Center, New York City, NY USA; 9 Department of Radiology, Kananzawa University, Japan, 2-21-9 Asahi-machi, Kanazawa, 920-0941 Japan; 10 Department of Radiology, Hospital Beaujon APHP.Nord, Université Paris Cité, CRI INSERM 1149, Paris, France; 11 University of California San Francisco, 505 Parnassus Avenue, Room M-361, San Francisco, CA 94143 USA

**Keywords:** Liver, Hepatocellular carcinoma, Ethiodized oil, Treatment response, Transarterial chemoembolization

## Abstract

**Abstract:**

Conventional transarterial chemoembolization (cTACE) utilizing ethiodized oil as a chemotherapy carrier has become a standard treatment for intermediate-stage hepatocellular carcinoma (HCC) and has been adopted as a bridging and downstaging therapy for liver transplantation. Water-in-oil emulsion made up of ethiodized oil and chemotherapy solution is retained in tumor vasculature resulting in high tissue drug concentration and low systemic chemotherapy doses. The density and distribution pattern of ethiodized oil within the tumor on post-treatment imaging are predictive of the extent of tumor necrosis and duration of response to treatment. This review describes the multiple roles of ethiodized oil, particularly in its role as a biomarker of tumor response to cTACE.

**Clinical relevance:**

With the increasing complexity of locoregional therapy options, including the use of combination therapies, treatment response assessment has become challenging; Ethiodized oil deposition patterns can serve as an imaging biomarker for the prediction of treatment response, and perhaps predict post-treatment prognosis.

**Key Points:**

*• Treatment response assessment after locoregional therapy to hepatocellular carcinoma is fraught with multiple challenges given the varied post-treatment imaging appearance.*

*• Ethiodized oil is unique in that its’ radiopacity can serve as an imaging biomarker to help predict treatment response.*

*• The pattern of deposition of ethiodozed oil has served as a mechanism to detect portions of tumor that are undertreated and can serve as an adjunct to enhancement in order to improve management in patients treated with intraarterial embolization with ethiodized oil.*

## Introduction

Hepatocellular carcinoma (HCC) is the third leading cause of cancer-related death worldwide, with a 5-year survival rate of 18% [1]. While curative treatment options include liver transplantation and surgical resection [2], more than 80% of patients have intermediate or advanced-stage disease at diagnosis, limiting treatment options to systemic therapy or intraarterial (IAT) locoregional treatment (LRT) such as transarterial chemoembolization (TACE), transarterial bland embolization (TAE), and transarterial radioembolization (TARE) [3]. TACE extends patient survival, palliates symptoms, prolongs time to progression, decreases the risk of progressing outside of Milan criteria to maintain liver transplant candidacy (bridge), and converts non-transplant candidates to transplant candidates (downstage) [3]. While staging plays an important role in the selection of the appropriate treatment options, ultimately, treatment decisions are made by a multidisciplinary tumor board and depend on various factors, including tumor location, size and multiplicity, disease stage, liver function, performance status, technical feasibility, potential for future transplant candidacy, and patient preference [4].

This manuscript focuses on ethiodized oil as an imaging biomarker for the prediction of TACE outcomes. Mechanism of action and clinical uses of ethiodized oil, post-treatment imaging appearance of ethiodized oil deposition patterns in tissue, and their predictive value for radiology-pathology correlation and survival are discussed. Guidance is provided for the use of ethiodized oil in clinical practice, and advanced diagnostic techniques employing ethiodized oil, including machine learning, are reviewed.

### Ethiodized oil is an integral part of cTACE

For cTACE procedures, ethiodized oil and ethiodized oil/drug emulsions demonstrate strong radio-opacity and non-miscibility with blood, and therefore, delivery can be monitored in real-time until the tumor vascular bed is saturated, and stasis of flow is achieved [5]. Ethiodized oil is a temporary embolic that can reach the portal venules around the tumor through the peribiliary plexus and can accumulate within small tumor deposits around the target lesion, which develop in the distribution of the small portal venule draining the tumor [6]. Opacification of small peripheral portal branches around the tumor with ethiodized oil is a common finding and has been demonstrated as a predictive factor for lower rate of local recurrence [6], and a higher rate of complete necrosis [7].

### Intraprocedural cone-beam CT as a predictor of response

Since ethiodized oil is highly radiopaque, it can be visualized on fluoroscopy and cone-beam CT (CBCT). CBCT is used worldwide interprocedurally during treatment with TACE to detect tumors and feeding arteries and to monitor the extent of embolization.

CBCT immediately after cTACE may predict local response. Iwazawa et al compared the diagnostic performance of intraprocedural CBCT and unenhanced CT performed 1 week after TACE for the depiction of intratumoral ethiodized oil accumulation. Sensitivity, specificity, and positive and negative predictive values were similar: 81% vs. 86%, 74% vs. 75%, 48% vs. 50%, and 93% vs. 95%, respectively (*p* = 0.449) [8]. This study demonstrated that CBCT has the intraprocedural capability to detect residual untreated disease, thus allowing for tumor-feeding arteries to be identified and treated. Adoption of intraprocedural CBCT may also obviate the need for follow-up CT one week following cTACE. Miyayama et al reported that intraprocedural monitoring of the embolized area by CBCT is able to improve the technical success of TACE (*p* < 0.001) and reduce local tumor progression (LTP) compared with TACE performed by digital subtraction angiography (DSA) alone in HCC lesions ≤ 6 cm. LTP rates at 1, 2, and 3 years after TACE were 22% vs. 33%, 27% vs. 41%, and 31% vs. 48% for intraprocedural CBCT and DSA, respectively [*p* = 0.0217]) [9].

### Imaging findings following cTACE

**Fig. 1:**
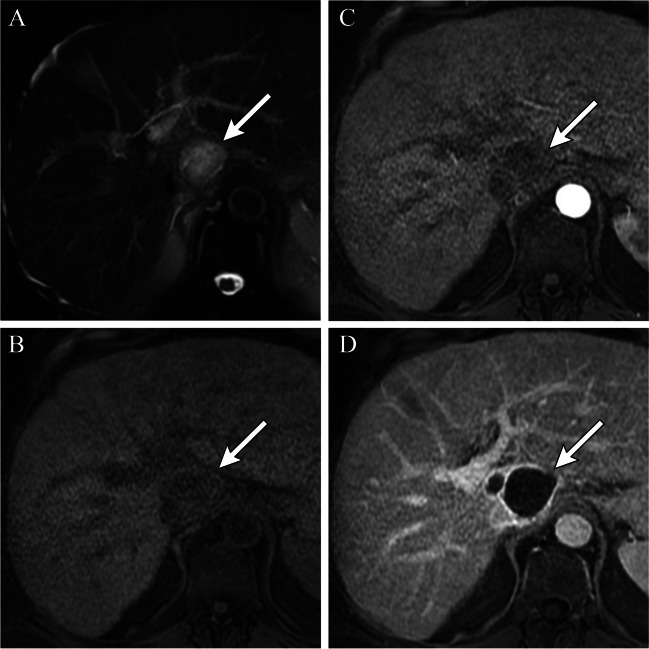
67-year-old male with HCV cirrhosis presenting with LR 5 HCC within the caudate lobe (images not shown). MRI post cTACE with lipiodol shows mild T2 hyperintense signal within the treated lesion (**A**). T1 pre-contrast fat-saturated images demonstrate hypointense signal within the treated lesion (**B**) with no post-contrast enhancement on arterial (**C**) or portal venous phase of imaging (**D**). Smooth perilesional rim of enhancement is consistent with expected post-treatment imaging findings (**D**). This lesion is categorized as mRECIST CR or LR-TR nonviable

After HCC treatment with cTACE there are expected treatment-specific imaging findings. cTACE creates ischemic and/or cytotoxic effects resulting in necrosis, with little to no change in tumor size early post-treatment and immediate non-enhancement [10] (Fig. 1). On MRI, post-treatment hemorrhage, inflammation, and/or liquefactive necrosis within the treatment zone [11] results in variable appearances on T1- and T2-weighted imaging [12]. Successfully treated HCCs usually demonstrate low signal intensity on T1- and T2-weighted images with no enhancement, unless there is hemorrhagic or proteinaceous debris, which may lead to high signal intensity on pre-contrast T1-weighted imaging [12]. In these cases, subtraction imaging can help identify subtle areas of residual arterial phase hyperenhancement (APHE), suggestive of viable tumor.

**Fig. 2:**
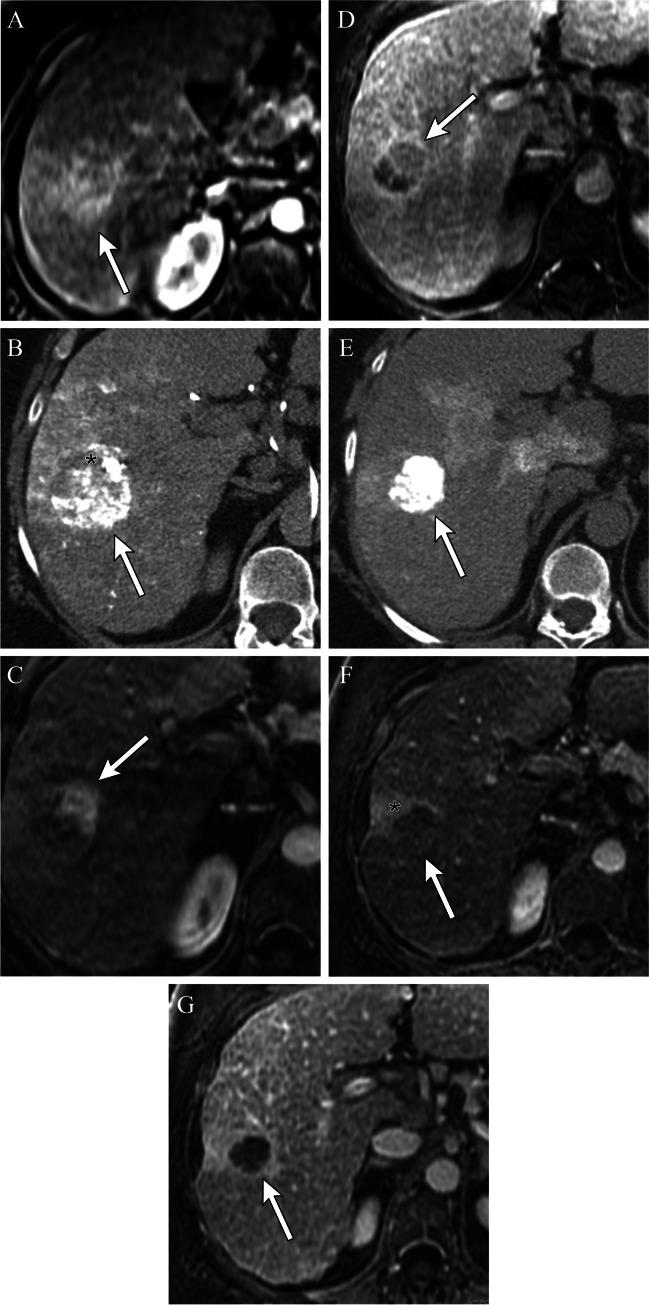
76-year-old female with a 3.8 cm LR 5 observation in segment 5/8 of the liver (**A**). Immediate post-Lipiodol TACE shows heterogeneous staining of the tumor with mild staining of the surrounding parenchyma (**B**). Arterial phase CT (**C**) performed 1 month after cTACE shows a thick nodular area of perilesional enhancement along the 12–3 o’clock margin of the treated tumor, with washout on a portal venous phase of imaging (**D**) compatible with viable disease, mRECIST PR or LR-TR Viable. Re-treatment with cTACE 1 month later shows dense homogeneous tumoral staining (**E**). One-month post-repeat cTACE shows no residual enhancement on arterial (**F**) or portal venous (**G**) phases of imaging. The treated lesion is now mRECIST CR or LR-TR Nonviable. Note the geographic perfusional changes in the parenchyma upstream from the tumor (**F**, **G**), an expected imaging feature. There is also a smooth perilesional rim of enhancement around the treated lesion (**G**), an expected imaging feature. The treated lesion is now mRECIST CR or LR-TR nonviable

A thin, smooth rim of APHE surrounding an effectively treated tumor after IAT, reflects inflammation [11] (Fig. 1). Locally recurrent or residual viable HCC presents as irregular, nodular areas of APHE or APHE plus washout within or along the margin of the treated tumor [12] (Figs. 1 and 2). T2- and diffusion-weighted sequences can help identify LTP, particularly in cases with equivocal findings. Short-term follow-up within 3 months allows for identification of growing regions of APHE or washout to confirm viable tumor.

**Fig. 3:**
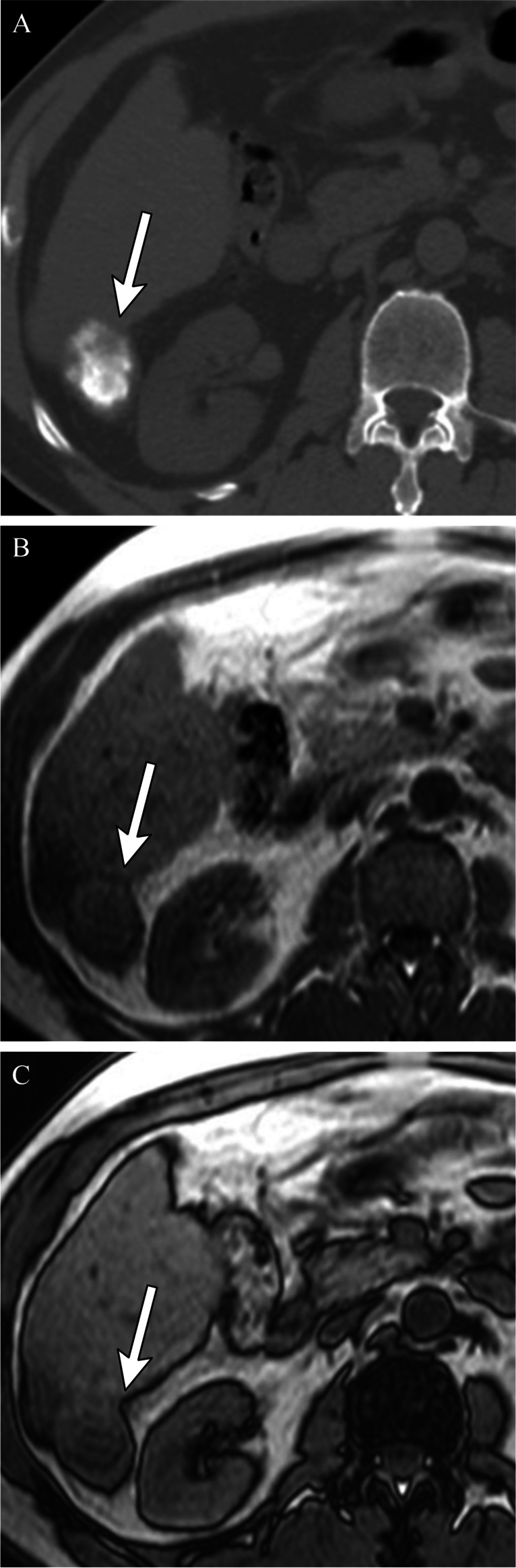
51-year-old male status post cTACE to a segment 6 LR 5 observation (images not shown). Non-contrast CT 1-month post-treatment shows Lipiodol staining of the targeted tumor (**A**). In (**B**) and opposed (**C**) phases MRI does not show the hyperdense appearance within the treatment cavity, and thus it is easier to detect areas of nodular viable tissue along the periphery of the treated lesion on MRI as compared to CT, where the Lipiodol hyperdensity may obscure subtle areas of peripheral nodular enhancement

**Fig. 4:**
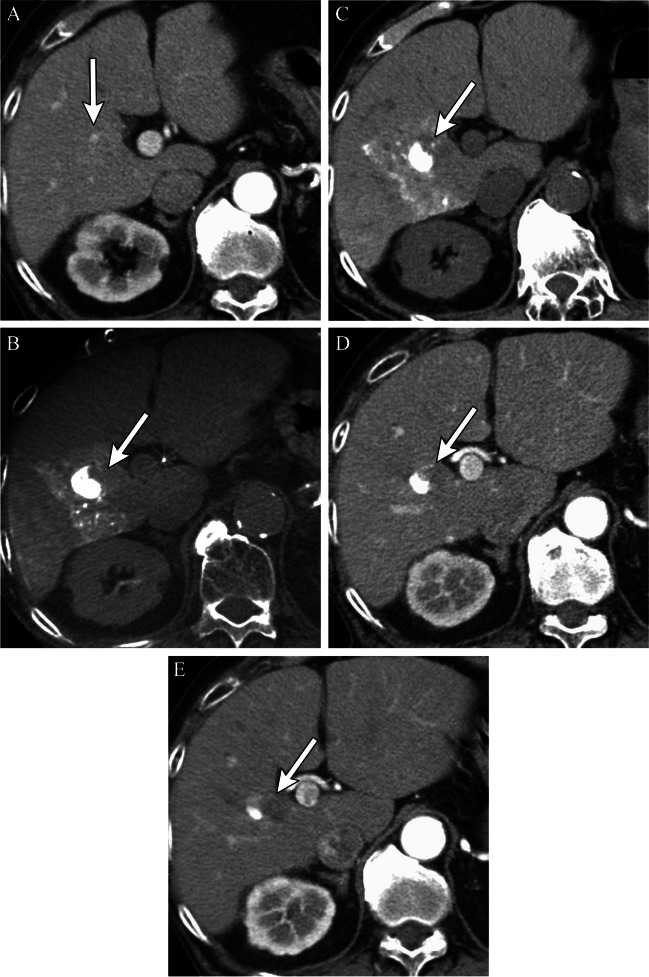
Defect of Lipiodol accumulation in the tumor on CBCT after TACE. 56-year-old with cirrhosis. Arterial-phase CT shows a small tumor (arrow) (**A**). TACE was performed, but CBCT was performed immediately after TACE demonstrated a defect of Lipiodol accumulation in the tumor. Another feeder was searched, but it could not be identified (**B**). Unenhanced CT performed 1 week after TACE also showed a defect of Lipiodol accumulation in the tumor (**C**). Arterial-phase CT performed 2 months after TACE revealed nodular APHE along the margin, compatible with viable tumor, LR-TR Viable (**D**). Five months post cTACE with no interval treatment, there is continued local progression with increasing size of the nodular perilesional tumor, LR-TR viable (**E**)

**Fig. 5:**
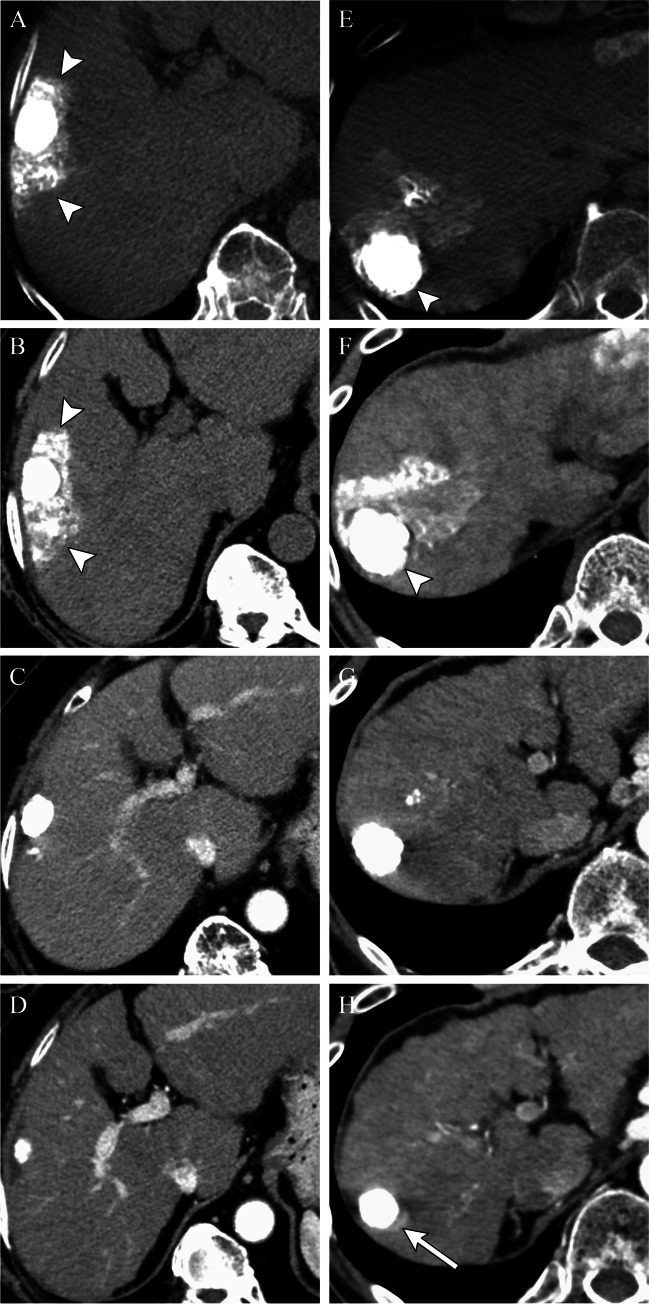
The necessity of Lipiodol accumulation around the tumor (a safety margin). Cirrhotic patient after cTACE for HCC. CBCT performed immediately after cTACE demonstrates that Lipiodol was densely accumulated in the entire tumor and moderately accumulated in the hepatic parenchyma surrounding the tumor with a safety margin (**A**). Unenhanced CT performed 1 week after cTACE showed complete embolization of the tumor (**B**). Arterial-phase CT performed 2 months after TACE showed complete response, mRECIST CR or LR-TR Nonviable (**C**), with excellent local control at 39 months (**D**). CBCT in another cirrhotic patient after cTACE for HCC shows homogenous dense Lipiodol staining in the entire tumor; however, the safety margin was not obtained along the posterior margin of the tumor (**E**). Non-contrast CT performed 1 week after TACE also shows the lack of the safety margin (**F**). Arterial-phase CT performed 2 months after cTACE shows persistent dense staining of the tumor with no evidence of abnormal APHE to suggest local recurrence, mRECIST CR, or LR-TR Nonviable (**G**). However, contrast-enhanced CT 12 months after cTACE shows local tumor recurrence where the safety margin was not obtained, as manifest by crescentic arterial phase hyperenhancement posterior to the treated lesion (**H**)

**Fig. 6:**
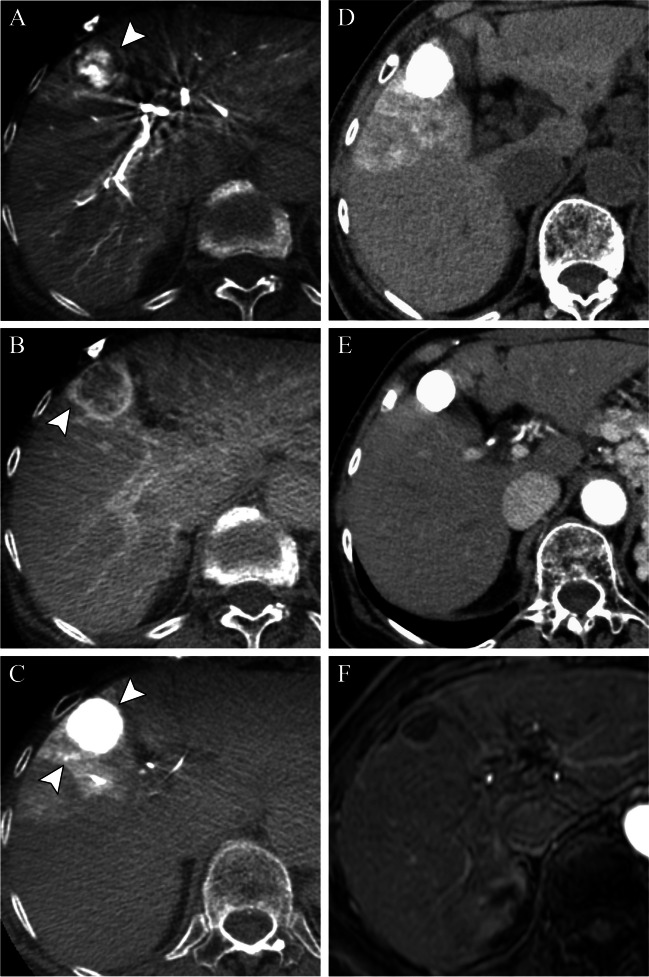
Corona enhancement on 2^nd^-phase CBCT during hepatic arteriography. On pre-treatment arterial phase CBCT during hepatic arteriography (CBCTHA), the central aspect of the targeted tumor showed hypervascularity with a circumferential peripheral rim hypovascular tumor (**A**). Corona enhancement was clearly demonstrated on portal venous phase CBCTHA (**B**). CBCT performed immediately after TACE demonstrated that Lipiodol was densely and uniformly accumulated in the tumor including within the hypovascular rim, as well as staining of the corona enhancement area (**C**). Unenhanced CT performed 1 week after cTACE shows complete embolization of the tumor (**D**). Arterial-phase CT performed 2 months after TACE shows mRECIST CR/LR-TR Nonviable of the tumor (**E**), and arterial-phase MRI performed 4 years after TACE also shows no tumor recurrence, with interval decrease in size and persistent smooth peripheral rim of enhancement, an expected post-TACE imaging feature (**F**)

A unique post-treatment feature is the hyperdense CT appearance of the treatment zone secondary to iodized oil deposition (Figs. 1, 2, 3, 4, 5, and 6). This hyperdensity limits assessment for tumor viability on post-contrast CT, as subtle areas of peripheral APHE cannot be discerned from the hyperdense iodized oil. MRI can better detect subtle areas of tumor viability, as the microfatty composition of the ethiodized oil results in loss of signal on out-of-phase imaging when compared to in-phase imaging, and is not seen on T1 fat-suppressed post-contrast images; thus MRI may improve treatment response assessment after cTACE [13] (Fig. 3).

After cTACE, there can be geographic parenchymal APHE secondary to the embolic treatment effect. Iso or mild hyperenhancement of the involved parenchyma, as compared to the remaining hepatic parenchyma, usually persists on portal venous and delayed imaging, without washout (Fig. 4). This imaging feature helps to differentiate the enhancement of viable tumors from perfusional changes secondary to inflammation and arterial embolization, in which case there is usually washout associated with the arterially enhancing viable tumor [14]. Over time, perfusional changes tend to regress and resolve.

### Ethiodized oil as a predictor of response on post-treatment CT

When ethiodized oil is administered via the hepatic artery, it distributes in tumor tissue and the surrounding liver parenchyma in proportion to the degree of tissue vascularity [15]. Traditionally, imaging evaluation of treatment response assessment after cTACE is performed within the first month after treatment, not only for the evaluation of the technical success of TACE for target tumors but also for the detection of daughter nodules [16]. Since the presence of hyperdense ethiodized oil could mask residual enhancing, viable tumors on CT imaging, the amount and pattern of distribution of ethiodized oil may serve as possible imaging biomarkers of tumor response, which may predict patient outcomes.

At the time of treatment, ethiodized oil is concentrated in necrotic tumor tissue and can be retained for a variable period of time depending on the degree of resultant tumor necrosis [15, 17]. The association between ethiodized oil deposition and tumor response may be explained by the fact that the portion of the tumor containing ethiodized oil is usually necrotic on pathological examination [18]. A recent prospective study of 39 patients (with both HCC and non-HCC malignancies) evaluated the correlation between ethiodized oil deposition at 24 h post-TACE CT and tumor response at 30 days [19]. In this study patients demonstrating tumor response were characterized by higher tumor ethiodized oil deposition.

Tumor recurrence may be detected when there is a defect in ethiodized oil accumulation within a tumor [16] (Figs. 2 and 4). Studies show that the presence of ethiodized oil deposition in at least 75% of the volume of the target lesion can be a predictor of improved survival. In a retrospective cohort of 85 histologically proven HCC, 1-year survival rate was 97% when this threshold was achieved, but drastically decreased if ethiodized oil retention was less than expected (1-year survival rate of 50% and 28% when ethiodized oil deposition was  < 75% and  < 50%, respectively) [20]. Similarly, another study showed that the presence of poor ethiodized oil deposition may predict a shorter time to progression and overall survival [21], while another study confirmed that a low ethiodized oil uptake and higher washout rate after TACE were significant predictors of early tumor recurrence for HCC [22]. A threshold of ethiodized oil uptake of 270 HU was associated with improved response [22].

In addition to the amount of ethiodized oil retention, the deposition pattern may be used as an imaging biomarker of tumor response. Dioguardi et al showed that for patients who underwent TACE before liver transplantation for HCC, the rate of tumor necrosis of nodules classified as having achieved complete response according to mRECIST criteria, was higher when the tumor had a complete ethiodized oil deposition pattern. In this study, the rate of complete pathologic necrosis was found to be 95% for tumor nodules with complete ethiodized oil deposition and 65% for lesions with incomplete ethiodized oil deposition [23].

Another study classified ethiodized oil retention patterns in tumors as complete (covering the entire tumor volume), or incomplete [24] (Figs. 2, 3, and 4). Local progression was defined as the reappearance of areas of enhancement on arterial phase images with washout on portal or delayed phase images within 2 cm from the treated tumors on follow-up imaging studies. A total of 46 (56%) HCC lesions were deemed to have a complete radiographic response by mRECIST, of which 16 (35%) had incomplete and 30 (56%) had complete ethiodized oil coverage. After a median follow-up of 14 months, 15/16 (94%) and 10/30 (30%) of incompletely and completely -stained lesions developed local progression, respectively (*p* < 0.001). No significant difference in time to progression was detected with respect to the extent of ethiodized oil coverage (mean 11 vs 13 months for incomplete and complete ethiodized oil coverage) [24]. Thus, the extent of ethiodized oil distribution within a lesion may help predict treatment response. Incomplete ethiodized oil distribution within a tumor staining could prompt additional treatment such as thermal ablation or repeat IAT without waiting for radiographic evidence of local disease progression.

Similarly, a series of 490 patients with unresectable HCC showed that the presence of complete ethiodized oil deposition at CT 1 month following TACE was associated with improved survival [25]. In this study, 1, 3, and 5-year survival rates were 92.7%, 70.7%, and 52.4% for patients with tumors showing complete ethiodized oil deposition vs 60.8%, 28.0%, and 16.9% with incomplete ethiodized oil deposition, respectively. This study also showed that complete ethiodized oil deposition after TACE was observed more frequently in patients with single and small (< 5 cm) HCCs [25].

### Treatment zone margins

The need for a safety margin for cTACE remains controversial [26], even though it is an established concept for other treatment options, such as surgical resection and thermal ablation. Histologically, blood exits HCC tumors into the peritumoral portal venules through the tumor capsule and into surrounding hepatic sinusoids in non-capsulated HCC [27, 28]. The drainage area can be seen as corona enhancement around the tumor on the delayed phase of CBCT during hepatic arteriography and should be included in the treatment area because it is a high-risk area for the development of intrahepatic metastases [27, 29, 30] (Figs. 5 and 6). A distinct advantage of iodized oil for IAT is its ability to pass through these tumor drainage channels and temporarily block the portal venules and sinusoids in the corona area, presumably treating disease below the threshold of radiographic detection, translating into longer disease-free and overall survival [31]. The width of corona enhancement depends on tumor vascularity and may vary at different tumor regions [27, 28]. Adequate 3D-safety margins of lipiodol deposition, defined as lipiodol accumulation in liver parenchyma equal to or more than one millimeter surrounding the tumor, has shown to be associated with higher local disease-free survival (82.8% vs 19.3%). Moreover, for patients newly diagnosed with HCC, the median overall survival was significantly higher when adequate margins were present [32]. Presumably, adjacent parenchymal staining with lipiodol helps to treat disease below the threshold of radiographic detection, thus translating into longer disease-free and overall survival.

Studies have shown that LTP develops more frequently in tumors embolized without a safety margin than tumors with a safety margin (*p* = 0.0016) and intrahepatic distant recurrences (IDR) also develop more frequently in patients with LTP (*p* = 0.0004) [33]. A recent study demonstrated radiographic complete response rates at 1 and 3 months of 84% vs. 36% and 75% vs. 28% for cTACE and drug-eluting beads TACE (DEB-TACE), respectively (*p* < 0.0001) [34]. Given these data, it can be hypothesized that cTACE may confer an advantage in local tumor control over DEB-TACE because of its ability to embolize the tiny peri-tumoral venules which are a route for micrometastasis.

## Ethiodized oil as a predictor of pathologic response to cTACE

The experience with correlating imaging features of HCC lesions following cTACE with findings at pathology and clinical outcomes has been limited [18, 35–38] since only a minority of patients are candidates for resection or liver transplantation. Nevertheless, studies suggest that dense tumor stains with ethiodized oil [37] and lack of residual tumor enhancement on post-TACE CT or MRI [18, 36, 38] are associated with greater tumor necrosis at pathology and longer disease-free survival.

In a study with 132 cTACE-treated HCC lesions with eventual liver transplantation, near-complete lesion necrosis was associated with extensive lipiodol accumulation within a lesion during TACE administration (*p* = 0.02). On post-TACE computed tomography, lack of residual contrast enhancement (*p* < 0.0001), decrease in lesion size (*p* = 0.009), high lesion density due to lipiodol accumulation (*p* = 0.005), and diffuse distribution of lipiodol throughout the lesion (*p* < 0.0001) were also correlated with near-complete lesion necrosis at pathology [39].

### Parametric response mapping after intraarterial therapy

Parametric response mapping (PRM) is a novel voxel-based imaging tool that provides a semi-automated and quantitative assessment of tumor viability that can be used to assess HCC following cTACE [40]. PRM provides information about tumor viability by comparing density values on arterial and portal venous phase (PVP) images within a region of interest (ROI) on both pre- and post-treatment scans [41]. To allow for precise calculation, PVP images are spatially deformed to match the arterial phase images for correct registration. Following the registration process, the target lesion is segmented, from which a voxel-based density distribution is generated using separate software. Utilizing the segmentation data, a scatter plot is generated and different threshold values are subsequently assigned to define tumor components. For example, voxels with an arterial and PVP density of 0–30 HU are defined as necrosis, voxels greater than 300 HU are described as calcification/lipoidol, and voxels ranging between 30 and 300 HU are categorized as viable tumors.

Several studies have reported on tumor response assessment utilizing PRM [42]. In 2014, Choi et al [41] reported the feasibility of PRM to detect viable tumors in HCC treated with cTACE by assessing 35 ethiodized oil defect areas (IODA). Patients were divided into a viable (*n* = 22) and non-viable group (*n* = 13) based on the presence of IODA. Comparison was made between manual analysis (done by radiologists), PRM results, and combining PRM results with an automatic classifier to distinguish between two tumor groups based on dynamic CT images from two longitudinal exams. Areas under the curve (AUC) were compared amongst the three groups: 0.74 versus 0.84 versus 0.87 for manual versus PRM versus PRM with an automatic classifier, showing the highest yield with utilization of PRM with an automatic classifier [41].

In another study, PRM was useful in predicting HCC recurrence following cTACE by extrapolating data from longitudinally acquired CT exams, leading to the development of an algorithm for early prediction of cTACE outcomes [43]. Manual analysis was compared to PRM analysis with an AUC of 0.64 to 0.76, suggesting that PRM analysis may be superior. This allowed for individualized treatment with short-term follow-up plans for suspected recurrence in the absence of radiological signs of recurrence. Additionally, PRM has proven to be a better predictor of overall survival for HCC patients undergoing TACE than conventional imaging biomarkers [44].

Despite promising results, the relatively small sample size of these studies is a barrier to the adoption of PRM for HCC treatment assessment. Further research studies evaluating the accuracy of PRM for HCC treatment response assessment in comparison with established imaging techniques, such as dynamic contrast-enhanced MRI, are needed.

### Machine learning as a predictor of response

Prognostic information can be extracted from dynamic contrast-enhanced CT and MRI examinations obtained for HCC diagnosis and treatment follow-up without incurring additional cost, time, or radiation dose to the patients. Some studies have developed prognostic scoring systems for which patients should undergo TACE as initial treatment, which take account into laboratory results and simple imaging findings [45, 46]. However, conventional image interpretation has inherent pitfalls, such as subjectivity, inter-observer variation, and the inability to quantitatively analyze all the imaging findings.

Radiomics is a data-centric field that quantifies tumor imaging phenotypes through the extraction and mining of quantitative features [47]. It assumes that the image phenotype represents the underlying pathophysiology and therefore provides valuable information for tumor diagnosis and prognosis prediction [48]. Traditional radiomics studies rely on explicitly programmed algorithms to extract engineered (hand-crafted) imaging features such as tumor shape, voxel intensity information (statistics), and patterns (texture). Machine learning algorithms and feature selection [49] are then used to extract highly predictive imaging phenotypes and construct predictive models. Compared with traditional machine learning, deep learning (DL) does not require manual feature extraction. DL models can automatically learn which features should be extracted from the training set data. This eliminates human subjectivity in the model-building process and extracts richer and deeper features. Due to excellent visualization across all cross-sectional x-ray-based imaging modalities, ethiodized oil retained in target lesions after TACE has been successfully established as a theranostic imaging biomarker for the prediction of tumor response [42, 43]. The pattern of oil deposits is known to reflect tumor viability and can therefore be analyzed using advanced image analysis instruments. In this context, significant efforts are underway to utilize radiomics and deep learning techniques to take advantage of the imaging appearance of ethiodized oil both in directly intra-procedurally acquired imaging as well as in periprocedural cross-sectional imaging.

Current studies have mainly focused on radiomics, where a series of traditional machine learning models use pretreatment imaging and clinical data to predict outcomes after initial cTACE treatment for HCC. Sun et al used radiomics features from MRI to predict progression after TACE for Barcelona Clinic Liver Cancer (BCLC) B stage HCC. The model based on DWI features achieved an AUC of 0.786 and 0.729 [50], while the model based on T2WI features and clinical factors achieved an AUC of 0.813 in predicting response regardless of tumor stage [51]. The combined model including radiomics features from MRI or CT and clinical factors performed better than the clinical model and radiomics model alone with respect to prediction of survival and response to cTACE. Dong et al [52] compared six different machine learning models for predicting early response to cTACE, with random forest (RF) having the highest AUC. For patients undergoing transplantation, the pre-TACE radiomics model based on CT also demonstrated potential in the prediction of outcome [53]. The clinical-radiomics nomograms achieved a C-index of 0.833 and 0.739 in predicting overall survival and time to progression in advanced HCC patients treated with TACE plus Apatinib [54]. Radiomic features from non-contrast CT have also been shown to predict TACE response, with the highest AUC of 0.840 [55].

In addition to radiomics, studies have proposed DL models for outcome prediction based on MRI, CT, or digital subtraction angiography. Pan et al trained a convolutional neural network model based on the ResNet architecture on MDCT images that achieved an AUC of 0.82 for progression prediction in patients treated with liver resection or TACE [56]. The integrated nomogram with DL and clinical features showed significantly better prediction performance in overall survival than the clinical nomogram for patients treated with TACE plus sorafenib [57]. The prognostic model based on DL-score and clinical variables also demonstrated good accuracy in predicting the long-term survival of patients treated with TACE [58]. Both Tian et al [59] and Peng et al [60] developed models integrating DL features and radiomics features from MRI for predicting response after TACE treatment, with the highest AUC of 0.947 and 0.994. Although patients with HCC often have a background of cirrhosis, increasing the difficulty for algorithms to distinguish tumor from background liver parenchyma, Li et al [61] developed a CT-based multi-DL model which achieved the highest AUC of 0.871 in objective response prediction and the highest dice coefficient of 0.74 in tumor segmentation. Zhang et al [62] developed a DSA-based DL model that achieved an AUC of 0.78 in response prediction and the highest dice coefficient of 0.75 in tumor segmentation for the internal validation cohort, and 0.67 and 0.73 for the external validation cohorts.

Unfortunately, most studies are retrospective, only a few underwent external independent validation, and there is heterogeneity in the HCC stage and a lack of clinical interpretability for the features. In the future, multi-center data collection, inclusion of more clinical variables (e.g., tumor stage), and prospective studies before and after treatment will improve the robustness and increase the interpretability of machine learning models to quantify tumor characteristics, facilitate the construction of objective and reliable non-invasive markers for TACE efficacy assessment through machine learning, and promote the clinical translation of research results.

## Summary and conclusions

As clinical indications for IAT for HCC continue to expand, post-treatment response assessment is critical for management, including the need for repeat treatment, routine follow-up imaging, and evaluation for successful downstaging or bridging to transplant. Studies have shown the ability of ethiodized oil to act as an imaging biomarker to predict treatment response. Its radio-opacity allows for its use as an imaging biomarker to predict necrosis and outcomes in patients, as specific patterns of distribution, such as increased density of deposition, particularly in homogenous patterns, with a rim of deposition surrounding the radiographically visible margin of tumor in the normal parenchyma, are signs of excellent treatment response with lower rates of early post-treatment recurrence. Newer technologies such as cone beam CT, perfusion imaging, parametric response mapping, quantitative color mapping, and artificial intelligence such as machine learning are all novel techniques to help evaluate and predict treatment response after c TACE, with early promising results.

In conclusion, cTACE with ethiodized oil is an effective method of treatment for early and intermediate-stage HCC with excellent outcomes, including prolonged overall survival and time to recurrence. Ethiodized oil is unique in that its persistent visualization on post-treatment imaging allows it to serve as an imaging biomarker to predict response and prognosis.

## Abbreviations

AASLD: American Association for the Study of Liver Disease
APHE: Arterial phase hyperenhancement
BCLC: Barcelona Clinic Liver Cancer
CBCT: Cone-beam CT
cTACE: Conventional transarterial chemoembolization
DEB-TACE: Drug-eluting beads TACE
DL: Deep learning
DSA: Digital subtraction angiography
EASL: European Association for Study of the Liver criteria
HCC: Hepatocellular carcinoma
IAT: Intraarterial therapies
IDR: Intrahepatic distant recurrences
IODA: Oil defect areas
IRE: Irreversible electroporation
LRT: Locoregional treatment
MWA: Microwave
PBV: Parenchymal blood volume
PEI: Percutaneous ethanol injection
PRM: Parametric response mapping
qEASL: Quantitative European Association for Study of the Liver criteria
RCT: Randomized control trial
RF: Random forest
SBRT: Stereotactic body radiotherapy
TACE: Transarterial chemoembolization
TAE: Transarterial bland embolization
TARE: Transarterial radioembolization
TRA: LI-RADS Treatment Response Algorithm

## Declarations

### Guarantor

The scientific guarantor of this publication is Mishal Mendiratta-Lala MD.

### Statistics and biometry

No complex statistical methods were necessary for this paper.

### Study subjects or cohorts overlap

No.

### Methodology

• review
